# The mediation effects of coping style on the relationship between social support and anxiety in Chinese medical staff during COVID-19

**DOI:** 10.1186/s12913-020-05871-6

**Published:** 2020-11-04

**Authors:** Wei Zhu, Yi Wei, Xiandong Meng, Jiping Li

**Affiliations:** West China Hospital, Sichuan University, Chengdu City, Sichuan Province China

**Keywords:** Anxiety, Social support, Coping style, Mediation effect, COVID-19

## Abstract

**Background:**

The COVID-19 has been a pandemic around the world, which affirmatively brought mental health problems to medical staff. We aimed to investigate the prevalence of anxiety in Chinese medical staff and examine the mediation effects of coping styles on the relationship between social support and anxiety.

**Methods:**

A cross-sectional study via internet survey was conducted from 15 March to 30 March, 2020. The social demographic data, Self-rated Anxiety Scale, Social Support Rate Scale and Trait Coping Style Scale were collected. Pearson correlation and a structural equation model were performed to examine the relationships of these variables. The bootstrap analysis was conducted to evaluate the mediation effects.

**Results:**

A total of 453 medical staff participated in this study. The mean score of SAS was 46.1 (SD = 10.4). Up to 40.8% of the participants had anxiety symptoms. The participants lived with family members had lower SAS score (45.1 ± 9.8 vs 49.6 ± 11.8). Social support was negatively associated with anxiety, mediated by positive coping and negative coping partially significantly with an effect size of − 0.183.

**Conclusions:**

Chinese medical staff had a high level of anxiety during the COVID-19 pandemic. Coping styles had effects on the association between social support and anxiety. Sufficient social support and training on positive coping skills may reduce anxiety in medical staff.

**Supplementary Information:**

The online version contains supplementary material available at 10.1186/s12913-020-05871-6.

## Background

The coronavirus disease 2019 (COVID-19) first detected in Wuhan City has become a pandemic, threating public health worldwide [1, 2]. On March 31, over 750,000 confirmed cases and 36,000 deaths were reported worldwide by World Health Organization, of which over 82,000 confirmed cases and 3300 deaths were reported from China [3]. As the first-line responders treating COVID-19, medical staff face tough conditions, including a heavy workload, risk of infection, shortage of preventive equipment, separation from their social network and exposure to death frequently [4–6]. These conditions caused by COVID-19 epidemic affirmatively lead to mental health problems, such as anxiety, stress, and depression in medical staff [6, 7]. Of all mental health problems, anxiety of medical staff during the COVID-19 pandemic was reported as the main mental health concern with the highest prevalence based on a survey in China [5]. Anxiety was defined as an apprehension or dread feeling accompanied with varied autonomic symptoms [8]. Previous studies have found that people under high anxiety are more likely to have decreased energy and experience social isolation [9, 10], which may aggravate anxiety inversely. Additionally, a study on 180 medical staff treating COVID-19 by Xiao [11] revealed that anxiety had a negative association with sleep quality. Therefore, it is important to emphasize mental health, especially anxiety, in medical staff during the COVID-19 pandemic [7, 12, 13].

Social support is a critical approach to reduce anxiety. Social support generally refers to a belief that individuals are cared for, loved, esteemed and sharing the mutual obligation of a social network [14]. The friends, family members, colleagues, and even communities of individuals can provide social support from emotional, material and spiritual aspects [15, 16]. The positive effects of sufficient social support on anxiety has been proven in previous studies [17, 18]. Support from a social network provides a way for individuals to share negative or traumatic life events [19]. This interaction between individuals and their social supporters may bring empathy and emotional well-being, thus achieving a better mood [20, 21]. Additionally, Glozah [22] believed that adequate social support can lead to increased courage, a better interpersonal understanding and a sense of professional achievement by increasing self-efficacy. These positive emotional experiences provided by social support can effectively decrease anxiety.

Coping styles are also correlated with anxiety. When facing difficult or stressful life events, individuals will take different cognitive, emotional or behavioural response strategies, which are defined as coping styles [23]. These styles were divided into positive coping and negative coping by Jiang [24], of which positive coping meant a problem-solving strategy and negative coping meant an emotion-focused coping mechanism, even not coping [25]. Previous studies found that coping style was correlated with anxiety [26–28]: positive coping was associated with anxiety negatively; negative coping was associated with anxiety positively. There are some assumptions about the mechanism of coping style influencing anxiety. Classen [29] reported that individuals with a positive coping strategy usually had a fighting spirit and a better emotional expression performance, which was considered to indicate good psychological adjustment ability, leading to lower anxiety. Coping styles may also influence anxiety through individuals’ normal or pathological changes of biological levels [23].

Based on the associations between social support, coping styles and anxiety, the potential inter-relationship between social support and coping styles was also reported in some studies. Ren [30] conducted a survey in pregnant women after an earthquake, in which they found that negative coping style was significantly associated with a lower level of subjective social support, objective social support and support use. Furthermore, social support and coping styles were both associated with mental health disorders in pregnant women. Geng [31] found that coping styles counterbalanced the effect of social support on self-management behaviours, which can influence the mental health status [32]. Thus, it is reasonable to hypothesize that coping styles may counterbalance the relationship between social support and anxiety. Previous studies confirmed that social support, coping styles and anxiety were inter-related. However, research focused on mechanism was more important than on the associations between different variables. Therefore, we aimed to evaluate the anxiety level of medical staff during the COVID-19 pandemic and examine whether coping styles mediate the association between social support and anxiety.

## Methods

### Participants and procedure

This cross-sectional study was performed in Chinese medical staff who worked during the outbreak of COVID-19 in Sichuan Province via an internet survey from March 15 to March 30, 2020. The questionnaire (Additional file 1) was built on a network platform (www.wjx.cn) and then was shared on social media including *WeChat* and *Tencent QQ*. A convenience sampling method was used to recruit participants in this study. The inclusion criteria were as follows: 18 years old or above; regular employees; worked at their posts rather than engaged in advanced studies or went on a business trip during the survey. After a brief written informed consent at the beginning of the survey, three questionnaires about social support, anxiety symptoms and coping styles were required. Socio-demographic data including age, gender, marital status, living with family members or not, employee type and seniority, were also required. While constructing the online questionnaire, the integrity check function of the platform was used, meaning the questionnaire could not be submitted unless all questions were answered. After extracting the data from the platform, two researchers re-checked the quality of the questionnaires to eliminate those with missing data independently until a 100% consensus was reached.

### Measures

Coping styles: The Trait Coping Style Questionnaire (TCSQ) developed by Jiang [33] was used to evaluate the coping styles of Chinese medical staff. This questionnaire consists of two dimensions (positive coping and negative coping) with 20 items and uses a five-point Likert-scale, where 1 means “absolutely not” and 5 means “absolutely yes”. The total score of each dimension ranges from 10 to 50, with higher scores indicating that someone may be more likely to utilize a positive or negative coping style. The Cronbach’s alpha value of positive and negative coping dimensions was 0.70 and 0.69, respectively [33]. In this study, the Cronbach alpha coefficient was 0.84.

Social support: The Chinese Social Support Rate Scale developed by Xiao [34] was used to assess social support. This questionnaire contains 10 items, which are divided into three dimensions, including subjective support (items 1, 3, 4, 5), objective support (items 2, 6, 7) and availability (items 8, 9, 10). The score of items 1, 2, 3, 4, 8, 9 and 10 ranges from 1 to 4. Item 5 consists of 4 choices, where the total score is the sum of each choice ranges from 1 (never) to 4 (always). The score of items 6 and 7 is the count of the choice. A higher total score indicates better social support. The Cronbach’s alpha of this scale value was 0.76 [35]. In this study, the Cronbach alpha coefficient was 0.66.

Anxiety symptoms: The Self-rated Anxiety Scale developed by Zung [36] was used to evaluate anxiety symptoms. It is a four-point Likert-scale with 20 items. The standard total score ranges from 25 to 100, which is converted from the original score by multiplying by 1.25. A higher score indicates a higher level of anxiety. A score of 50–59 is classified as mild anxiety; a score of 60–69 is classified as moderate anxiety; and a score over 69 is classified as severe anxiety. This scale was proved good reliability and validity. The Cronbach alpha value in Chinese population was 0.76 [37]. In this study, the Cronbach alpha coefficient was 0.80.

### Data analysis

Socio-demographic variables were described using appropriate methods. Pearson correlation and multiple liner regression were performed to examine the relationships between social support, anxiety, and coping styles. These analyses were conducted in SPSS 23.0 (SPSS Inc., Chicago, USA, IL), with a significant *p* value of 0.05. Then, a structural equational model was conducted to assess the hypothesized mediation model using AMOS 24.0 (SPSS Inc., Chicago, USA, IL). The root-mean-square-error of approximation (RMSEA), goodness-of-fit index (GFI), adjusted goodness-of-fit index (AGFI) and Akaike Information Criterion (AIC) were used to evaluate the optimum model. The process macro provided by Hayes [38] was used to perform the bootstrap analysis to examine the mediation effects with 1000 bootstrap samples. The mediation effects were considered significant if the confidence intervals did not include the value of zero [39].

#### Ethic consideration

This study was approved by the Ethics Committee of West China Hospital, Sichuan University (ID: 2020–254). We provided an online written informed consent at the beginning of the online questionnaire. The survey started after the participants agreed to participate in this study and filled out the informed consents. All participants were informed that they had the right to withdraw from this study at any time.

## Results

### Socio-demographic characteristics and anxiety level of the participants

A total of 453 Chinese medical staff participated in this study with 94.9% female and 5.1% male participants (Table 1). Of the participants, nurses were the largest proportion (87.4%). The participants in this study were mainly individuals under 45 years old (85.4%), and 77.7% of participants lived with their family members during the COVID-19 outbreak. The mean score of SAS was 46.1 (SD = 10.4). A total of 185 (40.8%) participants showed anxiety symptoms. Of the participants with anxiety, 28.6% had moderate and severe anxiety. The SAS scores showed no significant differences regarding age, gender, marital status, employee type or seniority. The participants living with family members had lower SAS scores (45.1 ± 9.8 vs 49.6 ± 11.8, *p* < 0.001).

**Table 1:** The characteristics and anxiety level of participants (*n* = 453)

Variables	*n* (%)	SAS	F	*P* value
Age (years)				
18–34	281 (62.0)	46.5 ± 10.7	0.486	0.692
35–44	106 (23.4)	45.8 ± 10.0		
45–54	60 (13.2)	44.9 ± 9.5		
≥ 55	6 (1.4)	44.7 ± 14.5		
Gender				
Male	23 (5.1)	46.2 ± 8.6	0.001	0.979
Female	430 (94.9)	46.1 ± 10.5		
Marital status				
Married	317 (70.0)	45.6 ± 10.1	3.051	0.081
Single	136 (30.0)	47.4 ± 11.1		
Living with family members				
Yes	352 (77.7)	45.1 ± 9.8	14.666	< 0.001
No	101 (22.3)	49.6 ± 11.8		
Employee type				
Doctors	19 (4.2)	44.7 ± 9.5	1.067	0.363
Nurses	396 (87.4)	46.3 ± 10.6		
Medical technician	15 (3.3)	41.7 ± 7.3		
Workers	23 (5.1)	46.6 ± 9.4		
Seniority (years)				
0–5	141 (31.1)	46.3 ± 11.2	0.570	0.685
6–10	119 (26.3)	46.4 ± 10.0		
10–15	73 (16.1)	46.5 ± 10.6		
16–20	47 (10.4)	46.9 ± 10.1		
> 20	73 (16.1)	44.5 ± 9.6		

*SAS* Self-rated Anxiety Scale

### Correlation analysis between social support, coping styles and anxiety

Table 2 shows the mean, SD and correlations of the Social Support Rate Scale, Self-rated Anxiety Scale, Positive Coping and Negative Coping. The Social Support Rate Scale was negatively correlated with Negative Coping (*r* = − 0.283, *p* < 0.01) and Self-rated Anxiety Scale (*r* = − 0.294, *p* < 0.01); Social Support Rate Scale was positively correlated with Positive Coping (*r* = 0.146, *p* < 0.01). Negative Coping was positively correlated with Self-rated Anxiety Scale (*r* = 0.448, *p* < 0.01); Positive Coping was negatively correlated with Self-rated Anxiety Scale (*r* = − 0.237, *p* < 0.01). The multiple regression analysis indicated that social support and coping style were significantly related to anxiety symptoms, explaining 28.3% of all variance (Table 3).

**Table 2:** Correlations between anxiety symptom, trait coping styles and social support

Variables	SSRS	NC	PC	SAS
SSRS	1.0			
NC	− 0.283^a^	1.0		
PC	0.146^a^	0.061	1.0	
SAS	−0.294^a^	0.448^a^	−0.237^a^	1.0
Mean	41.2	28.2	38.1	46.1
SD	8.8	9.0	6.9	10.4

^a^: 0.01; *SSRS* Social Support Rate Scale, *NC* Negative coping, *PC* Positive coping, *SAS* Self-rated Anxiety Scale

**Table 3:** Regression analysis of the effects of social support and coping style on anxiety symptom

Variables	Std.β	95% CI	*P*	Adj.R^2^
NC	0.423	0.394, 0.584	< 0.001	0.283
PC	− 0.242	− 0.489, − 0.247	< 0.001	
SSRS	−0.139	− 0.262, − 0.067	0.001	

*NC* Negative coping, *PC* Positive coping, *SAS* Self-rated Anxiety Scale, *SSRS* Social Support Rate Scale

### Path analysis of effects of social support on anxiety

**Fig. 1:**
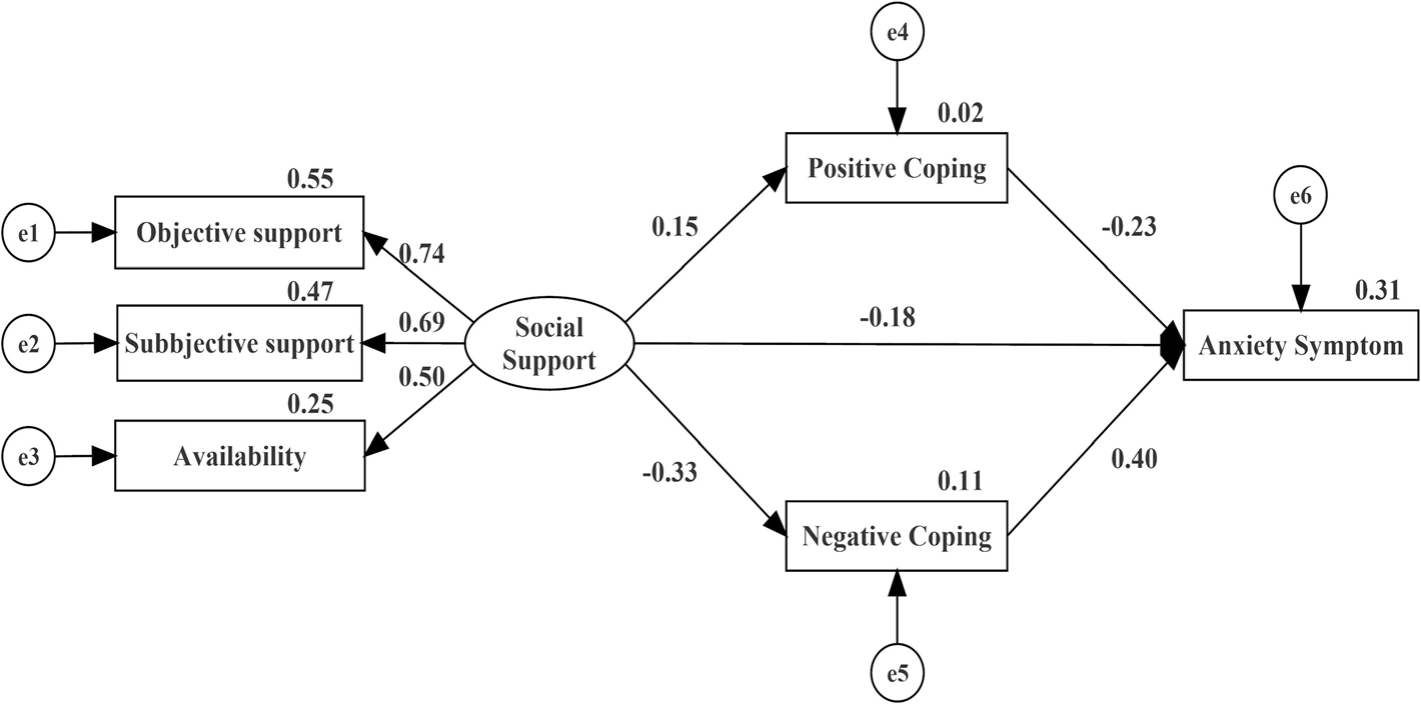
Path analysis of the social support, coping style and anxiety

The structural equation model (SEM) was conducted to measure the associations and importance of social support on anxiety in medical staff (Fig. 1). The fitness of the model was acceptable: χ^2^/df = 3.59, *p* < 0.01, GFI = 0.983, AGFI = 0.948, CFI = 0.958, RMSEA = 0.076, AIC = 53.159. The Social Support Rate Scale scores positively affected Positive Coping (*β* = 0.15, *p* = 0.007) and negatively affected Negative Coping (*β* = − 0.33, *p* < 0.001) and Self-rated Anxiety Scale scores (*β* = − 0.18, *p* < 0.001). Positive Coping negatively affected Self-rated Anxiety Scale scores (*β* = − 0.23, *p* < 0.001). Negative Coping positively affected Self-rated Anxiety Scale scores (*β* = 0.40, *p* < 0.001).

### Mediation effects of the coping style

The Social Support Rate Scale had a direct effect on the Self-rated Anxiety Scale. Additionally, the social support had indirect effects on the Self-rated Anxiety Scale through Positive Coping and Negative Coping paths, indicating the partial mediation effects of coping style. The results of bootstrap analysis are showed in Table 4. The 95% confidence intervals of the indirect effects of two different coping styles did not include the value of zero neither, showing significant differences.

**Table 4:** Bootstrap analysis of the mediation effect

Variables	Effect	SE	LL 95% CI	UL 95% CI
Direct effect	−0.164	0.050	−0.262	− 0.067
Indirect effect	−0.183	0.030	−0.241	− 0.126
SSRS-PC-SAS	−0.042	0.016	−0.076	− 0.013
SSRS-NC-SAS	−0.141	0.027	−0.192	− 0.091
Total effect	−0.347	0.053	−0.451	− 0.243

*NC* Negative coping, *PC* Positive coping, *SAS* Self-rated Anxiety Scale, *SSRS* Social Support Rate Scale

## Discussion

This study found that Chinese medical staff experienced a high level of anxiety. Sufficient social support can reduce anxiety directly. Meanwhile, social support affected anxiety through positive coping and negative coping paths indirectly.

This study found that Chinese medical staff working during the outbreak of COVID-19 had a higher level of anxiety compared to the Chinese medical staff norms (40.8% vs 31.0%) [8]. During the internet survey, the confirmed COVID-19 cases in China almost reached their highest level. The medical staff were required to finish routine treatment and prevent the infection of COVID-19 simultaneously, which may led to a higher workload and stress. Additionally, wearing protective equipment may lead to medical staff communicating with colleagues less frequently. These facts may contribute to a higher level of anxiety during the COVID-19 pandemic. The infection of medical staff also led to more anxiety [11]. Therefore, mental health care programmes for the medical staff are urgently needed. Chen [40] took a series of actions to maintain the mental health of their medical staff during the outbreak of COVID-19, which included setting up a rest area, providing food and information on the care of COVID-19 patients, etc. Liu [12] developed an online mental health service programme for medical staff during the COVID-19 pandemic, providing online counselling 24 h a day and all days of the week. However, the efficacy of the two programmes remains unknown.

It was not surprising to find that social support had a positive effect on anxiety directly. For medical staff, the story sharing and emotional expression to their friends and family members may lead to positive emotional experiences [41], resulting in decreased anxiety. Communication with colleagues is another form of receiving social support, giving medical staff the feeling of professional achievement and confidence in their work [42], reducing anxiety. This finding was consistent with previous results [18, 20]. Therefore, some measures can be adopted, such as setting up rest and communication aera, encouraging emotional expression and story sharing and providing mental health counselling services, to give sufficient social support for medical staff. Moreover, hospital managers can educate the family members on listening and empathy skills to help medical staff alleviate their anxiety.

This study also found that the relationship between social support and anxiety was partially mediated by coping styles. We demonstrated that positive coping strengthened the positive effect of social support on anxiety; negative coping negatively influenced the effect of social support on anxiety. In other words, when individuals received sufficient social support, they were more likely to use positive coping strategies to achieve a lower level of anxiety. If there was not enough social support, individuals tended to take negative actions facing problematic life events, resulting in higher anxiety. Additionally, if the level of social support remained stable or there was no way to provide more social support in short time, such as, the front-line medical staff living in a single room after work, providing positive coping skills may be helpful to reduce anxiety. Particularly, the total mediation effects of coping styles were 52.3%, suggesting an important role of coping style in the relationship between social support and anxiety.

In conclusion, healthcare workers are on the front line of the battle against COVID-19 and are paying the highest price for this global health emergency [43]. Protecting healthcare workers by providing psychological support and emotional skills to deal with anxiety is a priority [12, 44]. Sufficient social support affirmatively reduced the anxiety of healthcare workers. Thus, providing coping skills to healthcare workers may also contribute to decreasing anxiety when a coping style mediates the relationship between social support and anxiety.

## Limitations

There were some limitations in this study. First, this study was conducted through an internet survey, which may reduce the comprehensive understanding of the items of the scales. Second, the small samples were selected based on a convenience sampling method, thus the representativeness may be reduced. Third, the cross-sectional study cannot provide the causal relationships between these variables. A large sample-size and clinical review-based cohort study is needed in future.

## Conclusion

Chinese medical staff had a high level of anxiety during the COVID-19 pandemic. The mediation analysis found that coping styles affected on the association between social support and anxiety. Sufficient social support and training on positive coping skills may reduce anxiety in medical staff.

## Supplementary Information

**Additional file 1.**

## Data Availability

The datasets used and/or analyzed during the current study are available from the corresponding author on reasonable request.

## Abbreviations

COVID-19: Coronavirus disease 2019
TCSQ: Trait Coping Style Questionnaire
RMSEA: Root-mean-square-error of approximation
GFI: Goodness-of-fit index
AGFI: Adjusted goodness-of-fit index
AIC: Akaike Information Criterion
SSRS: Social Support Rate Scale
NC: Negative coping
PC: Positive coping
SAS: Self-rated Anxiety Scale
SD: Standard deviation
SEM: Structural equation model
