# Supplementary material for: The mediation effects of coping style on the relationship between social support and anxiety in Chinese medical staff during COVID-19
Source: BMC Health Serv Res. 2020 Nov 4;20:1007. doi: 10.1186/s12913-020-05871-6 (PMC7609823; doi:10.1186/s12913-020-05871-6)
Supplement: Supplementary file 1 — Additional file 1. [file 12913_2020_5871_MOESM1_ESM.docx]

Part 1. This part is about your socio-demographic situation. Please answer questions (1)-(6) below by selecting a choice.

(1) What is your age?

A.19-34 years old B. 35-44 years old C. 45-54 years old D. ≥55 years old

(2) What is your gender?

A. male B. Female C. Other___

(3) What is your marital status?

A. Single B. Married

(4) How about your living status?

A. Lived alone B. Lived with family members C. Other___

(5) What is your employee type?

A. Doctor B. Nurse C. Medical technician D. Worker

(6) How long have you worked in your institution?

A. 0-5 years B. 6-10 years C. 10-15 years D. 16-20 years E. >20 years

Part 2. This part is about your anxiety condition. Please answer questions (7)-(26) by selecting a choice which best describes how often you felt or behaved this way during the past 7 days (A. A little of the time B. Some of the time C. Good part of the time D. Most of the time).

(7) I feel more nervous and anxious than usual.

(8) I feel afraid for no reason at all.

(9) I get upset easily or feel panicky.

(10) I feel like I'm falling apart and going to pieces.

(11) I feel that everything is all right and nothing bad will happen.

(12) My arms and legs shake and tremble.

(13) I am bothered by headaches neck and back pain.

(14) I feel weak and get tired easily.

(15) I feel calm and can sit still easily.

(16) I can feel my heart beating fast.

(17) I am bothered by dizzy spells.

(18) I have fainting spells or feel like it.

(19) I can breathe in and out easily.

(20) I get feelings of numbness and tingling in my fingers & toes.

(21) I am bothered by stomach aches or indigestion.

(22) I must empty my bladder often.

(23) My hands are usually dry and warm.

(24) My face gets hot and blushes.

(25) I fall asleep easily and get a good night's rest.

(26) I have nightmares.

Part 3. This part is about your social support condition. Please answer questions (27)-(36) by selecting a choice which best describes your condition.

(27) How many close friends do you have that can get support or help?

A. 0 B. 1-2 C. 3-5 D. ≥6

(28) In the past year, ___

A. Away from family and live alone

B. Living quarters change frequently and spend most of the time with strangers

C. Live with classmates, colleagues, or friends

D. Live with family

(29) Regarding your neighbors, ___

A. Never cared about each other. You just know each other

B. You may be slightly concerned if confronting difficulties

C. Some of them care about you

D. Most of them care about you

(30) Regarding your colleagues, ___

A. Never cared about each other. You just know each other

B. You may be slightly concerned if confronting difficulties

C. Some of them care about you

D. Most of them care about you

(31) Support and care received from family members, ___ (1 . Never 2. A little 3. Some 4. Many)

1. Husband and wife (lovers) (1,2,3,4)

2. Parents (1,2,3,4)

3. Sons and daughters (1,2,3,4)

4. Brothers and sisters (1,2,3,4)

5. Others

(32) In case of emergency, you have received financial support or any help to solve practical problems from the following sources:

A. None

B. 1. spouse; 2. other family members; 3. relatives; 4. colleagues; 5. Institution; 6. official or semi-official organizations such as party; 7. non-imperial organizations such; 8. Others (Multiple choice)

(33) When you encountered emergency situations, you have received comfort and care from the following sources:

A. None

B. 1. spouse; 2. other family members; 3. relatives; 4. colleagues; 5. Institution; 6. official or semi-official organizations such as party; 7. non-imperial organizations such; 8. Others (Multiple choice)

(34) How to talk about your troubles?

A. Never complained to anyone.

B. Only talk to 1 or 2 individuals who are in a very close relationship.

C. You are willing to share if a friend asks

D. Talk your troubles positively to get support and understanding.

(35) The way to ask for help when you are in trouble

A. Rely only on yourself

B. Seldom ask for help

C. Sometimes ask for help

D. Often ask for help from family, friends, and organizations

(36) For group activity, ____

A. Never participate in

B. Sometimes participate in

C. Often participate in

D. Often participate in and be active in activities

Part 4. This part is about how you deal with difficulties or displeasure in your daily life. Please answer questions (37)-(56) by selecting a choice which best describes your condition. (1. Absolutely not 2. Sometimes not 3. Uncertain 4. Sometimes yes 5. Absolutely yes)

(37) I can forget the unhappiness as soon as possible

(38) I am easy to get caught up in memories and fantasies and can't get rid of them

(39) I can treat it as if it never happened

(40) I am apt to be angry with others and often loses his temper

(41) I usually look on the bright side

(42) Unpleasant things are easy to cause my mood swings

(43) I like to keep emotions in the bottom of my heart and not to share them. But I cannot forget them.

(44) I usually compared to others in similar conditions. Thus, it doesn't seem like a big deal

(45) I can quickly turn negative factors into positive factors by participating in activities

(46) I am easy to cry quietly

(47) Someone else can easily cheer me up again

(48) If I have a conflict with someone, I would rather ignore them for a long time

(49) I cannot decide easily or find a solution when confronting great difficulties

(50) I can adapt to difficulties and pain quickly

(51) I believe that difficulties and setbacks can make me stronger

(52) I am easy to recall unpleasant events in a long time

(53) When faced with problems, I often blame my own incompetence and resents myself

(54) I think there is nothing important in the world

(55) I like to be alone when facing difficulties

(56) I usually defuse an awkward situation with humor
